# Smoking status and its relationship with depression among the elderly population in Malaysia: Findings from the National Health and Morbidity Survey 2018

**DOI:** 10.18332/tid/169682

**Published:** 2023-08-30

**Authors:** Suthahar Ariaratnam, Cheong C. Kee, Ambigga D. Krishnapillai, Ridwan Sanaudi, Noorlaili Mohd Tohit, Kiau B. Ho, Sazlina Shariff Ghazali, Mohd Azahadi Omar

**Affiliations:** 1 Department of Psychiatry, Faculty of Medicine, Universiti Teknologi MARA (UiTM), Sungai Buloh, Malaysia; 2 Sector for Biostatistics and Data Repository, National Institutes of Health, Ministry of Health Malaysia, Shah Alam, Malaysia; 3 Department of Family Medicine, Faculty of Medicine and Defence Health, National Defence University of Malaysia, Kuala Lumpur, Malaysia; 4 Department of Family Medicine, Faculty of Medicine, Universiti Kebangsaan Malaysia, Kuala Lumpur, Malaysia; 5 Bandar Botanic Health Clinic, Klang, Malaysia; 6 Department of Family Medicine, Faculty of Medicine and Health Sciences, Universiti Putra Malaysia, Serdang, Malaysia

**Keywords:** elderly, smoking status, depression, National Health and Morbidity Survey

## Abstract

**INTRODUCTION:**

Literature exploring smoking status and its association with depression among the elderly population using nationwide data in Malaysia is limited. Hence, a nationwide survey to determine the prevalence of smoking and depression among the elderly (aged ≥60 years) population was undertaken.

**METHODS:**

This secondary dataset analysis used data from the National Health and Morbidity Survey (NHMS) 2018. Data from 3914 participants were collected on elderly health in the Malaysian population. Sociodemographic characteristics were recorded. Smoking status was grouped as current smokers, former smokers, and non-smokers. A validated Malay language version of the Geriatric Depression Scale (M-GDS-14) was used to screen for depression among the elderly.

**RESULTS:**

There was a significant association between smoking status with location, gender, employment status, marital status, ethnicity, education level, income, and depression. Current smokers are significantly higher in rural than urban areas. Among depressed participants, 65.7%, 17.1% and 17.2% were non-smokers, former smokers and current smokers, respectively. Multiple logistic regression showed that single (unmarried/separated/ divorced/widowed) participants were more likely to be depressed compared to married participants (AOR=1.68; 95% CI: 1.16–2.43). Whilst unemployed participants were more likely to be depressed than those who were employed (AOR=1.72; 95% CI: 1.22–2.44). Other Bumiputras were more likely to have depression compared to Malay, Chinese and Indian participants. Participants without formal education were more likely to be depressed compared to those having tertiary education. These participants have a 2-fold increased risk of depression (AOR=2.13; 95% CI: 1.02–4.45). Participants whose monthly salaries were <2000 MYR (AOR=3.67; 95% CI: 1.84–7.31) and 1000–1999 MYR (AOR=2.71; 95% CI: 1.23–5.94) were more likely to have depression compared with those who had received ≥3000 MYR. Ever smokers were more likely to be depressed than non-smokers (AOR=1.68; 95% CI: 1.23–2.29).

**CONCLUSIONS:**

Elderly Malaysians are indeed at risk of developing depression particularly if they had ever smoked. Public health awareness and campaigning are pertinent to disseminate these outcomes in order to spread the awareness associated with smoking-related depression.

## INTRODUCTION

With an ageing global population, identifying and comprehending the impact of smoking on mental health issues, particularly depression, in later life becomes pertinent not only for healthcare professionals but also for policymakers.

The National Health and Morbidity Survey for the year 2018 found that 11.2% of the elderly population reported having depressive symptoms^1^. In addition, this survey reported that elderly dwellers from rural areas were more likely to experience depression (14.4%) than those from urban areas (10.1%). Individuals who were single (never married/separated/divorced/widowed) (17.0%; 95% CI: 13.48–21.11) had more depressive symptoms than those who were married (8.6%; 95% CI: 7.14–10.40). The elderly people who were unemployed (unemployed/retiree/homemaker) had a higher predominance of depressive symptoms (12.7%; 95% CI: 10.58–15.13) compared to those who were employed. In regard to income, the elderly with the lowest income had the highest prevalence of depressive symptoms^2^.

Smoking among the elderly population has gained momentum recently, and as the proportion of the elderly population is expected to increase in the coming years, it is expected that there will be a rise in smoking-related diseases^3^. However, research examining the effect of smoking on depression in the elderly is limited^4^. According to a local study^5^, the prevalence of current smokers and ex-smokers among the elderly was 15.2% and 13.1%, respectively.

Among the elderly participants, previous studies had examined the link between smoking and chronic respiratory diseases^6^, diabetes and hypercholesterolemia^5^, dental caries and periodontitis^7^, quality of life^8^ as well as socioeconomic determinants^9^.

There is limited information on the basic mechanism linking smoking and depression. Smoking was described as having a pleasure-enhancing effect, an anxiety-lowering effect, and eliminating distress^10^. Anhedonia, anxiety sensitivity, and distress tolerance were three vulnerability factors implicated in smoking under the transdiagnostic emotional vulnerabilities framework. Additionally, this model proposes that anhedonia intensifies the pleasure-enhancing effect, anxiety sensitivity intensifies the anxiety-lowering effect, and poor distress tolerance increases distress elimination among smokers with depression or anxiety^10^.

Furthermore, a systematic review reported inconsistent evidence in the direction of the link between smoking and depression^11^. Depression or anxiety at baseline was associated with subsequent smoking behavior in almost half of the studies reviewed. This could be explained by the self-medication theory, which postulates that people with depression or anxiety smoke to alleviate their symptoms. Almost a third of the studies supported an alternative hypothesis, described as prolonged smoking putting individuals at higher susceptibility to environmental stressors, leading to depression or anxiety. Besides, there were few studies that reported a bi-directional link, while some studies found no association^11^.

There is a dearth of information investigating the association between smoking and depression among the elderly population on a national basis. Therefore, we undertook a nationwide survey to determine the association between smoking and depression among the elderly population in Malaysia.

## METHODS

This is a secondary analysis of data derived from the 2018 National Health and Morbidity Survey (NHMS) on the health of older adults, conducted by the Institute for Public Health, National Institutes of Health, Ministry of Health, Malaysia^1^. The NHMS 2018 was a cross-sectional, population-based, nationally representative survey, in which data collection was conducted between August 2018 and October 2018. The target populations of this survey were the pre-elderly aged 50–59 years, and the elderly aged ≥60 years. The Medical Research and Ethics Committee of the Ministry of Health Malaysia had approved this study (NMRR-17-2655-39047).

### Sampling method

The sampling frame of the survey was provided by the Department of Statistics of Malaysia, which was updated in 2017. Malaysia is geographically divided into about 83000 enumeration blocks (EBs). Each EB comprised approximately 80–120 living quarters (LQs), with an average household size of 4. This survey used multiple-stage cluster sampling. First, Malaysia was stratified into states. Second, each state was further stratified into urban and rural areas. The total number of EBs selected was proportionate to the population size. A total of 60 EBs from urban areas and 50 EBbs from rural areas were randomly selected, which consisted of 5636 LQs. All household members aged ≥50 years were recruited for the study. The detailed description for this survey is explained in the NHMS 2018 Methodology and General Findings report^1^.

### Study population

There were 3133 pre-elderly and 3959 elderly participants recruited for the survey. We included only 3914 elderly participants in the final analysis after excluding non-citizen and missing data.

### Data extraction

The data extracted from the NHMS 2018 were respondents’ sociodemographic characteristics, types of tobacco usage, and smoking status. The sociodemographic characteristics collected were the respondent’s current area of residence, gender, age, ethnicity, marital status, employment status, monthly income, and education level. The residential areas were divided into two categories: urban and rural areas. The ethnic groups comprised Malays, Chinese, Indians, other Bumiputras (which were Indigenous groups in Sabah and Sarawak), and other. Age was classified into 3 groups: 60–69 years, 70–79 years, and ≥80 years. The respondent’s marital status was categorized into two entities: married and single (unmarried/separated/divorced/widowed). The education level was divided into 4 levels: no formal education, primary education, secondary education, and tertiary education. The employment status was categorized as either employed or unemployed. Individual monthly income was classified into <1000, 1000–1999, 2000–2999, and ≥3000 MYR (100 Malaysian Ringgit about US$22). The smoking status was grouped as follows: ever smokers, which included present smokers who had currently used any smoked tobacco products daily or less than daily, former smokers who had used tobacco products in the past; and non-smokers who had never smoked in the past. A validated Malay language version of the Geriatric Depression Scale (M-GDS-14) was used for screening depression among the elderly^12^. The M-GDS-14 is an interviewer-administered questionnaire consisting of 14 questions requiring yes/no responses. For each question, a score of 1 is assigned for those who answered ‘yes’ and 0 for ‘no’, with the total score for M-GDS-14 ranging 0–14. A respondent is identified as having clinically significant depression^12^ if the M-GDS-14 scale score is ≥6.

### Statistical analysis

A weighting factor was applied to an individual’s data to adjust for non-response and probability of selection to account for the complex sample design and ensure a sufficient representation of the elderly population in Malaysia. The sociodemographic characteristics data (residential location, gender, age, ethnicity, marital status, education level, employment status, and monthly individual income received), and smoking status (non-smoker, former smoker, and current smoker) and their link to depression are presented as frequencies and percentages. The associations between sociodemographic characteristics, smoking status and depression were examined using Pearson’s chi-squared test. A univariable logistic regression was performed to assess the association between sociodemographic characteristics, smoking status, and the risk of depression. The variables with a significance level of p<0.25 were entered into a multivariable logistic regression analysis. Multiple variable logistic regression was applied to determine the association between smoking status and the risk of depression among the elderly while adjusting for other confounders such as residential location, gender, age, ethnicity, marital status, education level, employment status, and monthly individual income. The Hosmer-Lemeshow test was used to examine the goodness of fit of the model, and there were no significant 2-way interactions found among the variables. The classification table and receiver operator characteristic (ROC) curve were used to evaluate the predictive accuracy of the logistic regression model. All statistical analyses were carried out at the 95% significance level using IBM SPSS Statistics for Windows version 26.

## RESULTS

Table 1 depicts smoking status based on sociodemographic characteristics and depression profiles. There is a significant association between smoking status and residential location, gender, employment status, marital status, ethnicity, education level, income, and depression. Current smokers are significantly higher in rural areas than in urban areas. Among depressed participants, 65.7%, 17.1%, and 17.2% were non-smokers, former smokers, and current smokers, respectively.

**Table 1:** Sociodemographic characteristics and depression profiles based on smoking status (N=3914)

*Characteristics*	*Non-smokers (N=2797)*			*Former smokers (N=512)*			*Current smokers (N=605)*			p*
	*n*	*%*	*95% CI*	*n*	*%*	*95% CI*	*n*	*%*	*95% CI*	
**Location**										
Urban	1282	76.6	73.6–79.3	194	12.1	10.1–14.3	194	11.4	9.4–13.7	**<0.001**
Rural	1515	68.2	65.3–71.0	318	13.7	11.7–16.0	411	18.0	16.0–20.3	
**Gender**										
Male	839	51.3	47.0–55.6	443	23.4	20.3–26.8	555	25.3	22.1–28.7	**<0.001**
Female	1958	74.3	72.0–76.5	69	12.5	11.0–14.2	50	1.6	1.1–2.3	
**Age** (years)										
60–69	1808	73.9	71.3–76.3	288	12.0	10.2–14.1	422	14.1	12.2–16.3	0.08
70–79	787	76.2	72.4–79.5	157	12.3	10.1–14.9	148	11.6	9.2–14.4	
≥80	202	71.9	64.2–78.5	67	17.7	13.0–23.6	35	10.5	6.5–16.3.	
**Employment status**										
Employed	564	60.3	55.1–65.3	188	18.1	14.1–22.8	274	21.6	18.1–25.7	**<0.001**
Unemployed	2233	78.8	76.5–80.9	324	10.7	9.2–12.5	331	10.5	9.0–12.1	
**Marital status**										
Unmarried/separated/divorced/widowed	1076	84.1	81.0–86.8	104	6.0	4.6–7.9	146	9.9	7.7–12.5	**<0.001**
Married	1718	74.3	72.0–76.5	408	15.6	13.5–17.9	459	14.7	12.7–17.0	
**Ethnicity**										
Malays	1789	70.3	67.3–73.1	355	14.8	12.8–16.9	429	15.0	13.0–17.2	**0.001**
Chinese	564	81.5	77.4–84.9	68	9.0	6.6–12.2	71	9.5	7.2–12.4	
Indians	104	83.2	74.3–89.4	8	6.6	3.1–13.4	12	10.3	4.7–20.9	
Other Bumiputras	285	72.2	64.1–79.0	68	12.6	8.7–18.0	82	15.2	11.3–20.0	
Other	55	77.2	65.0–86.0	13	12.9	7.3–21.7	11	13.2	11.6–14.9	
**Education level**										
No formal education	605	80.7	76.8–84.0	94	9.6	7.3–12.6	88	9.7	7.5–12.4	**0.008**
Primary education	1288	70.8	67.5–73.8	267	13.4	11.4–15.7	348	15.9	13.8–18.2	
Secondary education	701	75.1	70.8–78.9	114	12.5	9.9–15.8	145	12.4	9.8–15.7	
Tertiary education	203	78.4	72.0–83.7	37	12.9	8.5–19.3	24	8.6	5.5–13.3	
**Income (MYR)**										
<1000	1826	77.1	74.2–79.7	277	9.9	8.2–11.9	369	13.0	11.2–15.1	**0.040**
1000–1999	541	68.3	63.8–72.5	138	16.2	13.0–20.1	157	15.5	12.6–19.0	
2000–2999	220	68.9	58.6–77.7	53	18.8	12.8–26.8	45	12.3	8.3–17.8	
≥3000	172	74.0	67.7–79.6	42	14.4	10.0–20.3	31	11.6	7.7–17.1	
**Depression**										
No	2354	75.3	73.1–77.5	400	12.0	10.3–13.8	491	12.7	11.1–14.6	**0.004**
Yes	304	65.7	60.6–70.5	81	17.1	17.2–13.2	88	17.2	13.2–22.2	

* Pearson’s chi-squared test.

Bold indicates statistical significance at p<0.05. MYR: 100 Malaysian Ringgit about US$22.

Multiple logistic regression (Table 2) shows that single (unmarried/separated/ divorced/widowed) participants were more likely to be depressed compared to married participants. In terms of employment status, unemployed participants were more likely to be depressed than those who were employed (adjusted odds ratio, AOR=1.72; 95% CI: 1.22–2.44). Ethnicity profiling showed that other bumiputras were more likely to have depression compared to Malays (AOR=0.67; 95% CI: 0.47–0.94), Chinese (AOR=0.41; 95% CI: 0.20–0.83), and Indian (AOR: 0.44; 95% CI: 0.19–1.00) participants. For education level status, the elderly who had no formal education were more likely to be depressed compared to those with tertiary education. Indeed, these participants had a 2-fold increased risk of depression (AOR= 2.13; 95% CI: 1.02–4.45). In the income category, participants with a monthly income of <2000 MYR (AOR=3.67; 95% CI: 1.84–7.31) and 1000–1999 MYR (AOR=2.71; 95% CI: 1.23–5.94) were more likely to have depression compared with those who had received ≥3000 MYR. In terms of smoking profile, ever smokers were more likely to be depressed than non-smokers (AOR=1.68; 95% CI: 1.23–2.29).

**Table 2:** Depression and its association with sociodemographic characteristics and smoking status among the elderly* (N=3684)

*Variable*	*OR (95% CI)*	*p*	*AOR (95% CI)*	*p*
**Location**				
Urban (Ref.)	1		1	
Rural	1.50 (1.06–2.13)	0.024	0.92 (0.60–1.4)	0.690
**Gender**				
Male (Ref.)	1		1	
Female	1.13 (0.90–1.42)	0.290	0.79 (0.55–1.13)	0.199
**Age (years)**				
60–69 (Ref.)	1		1	
70–79	3.04 (1.78–5.19)	<0.001	1.30 (0.91–1.87)	0.148
≥80	1.81 (1.31–2.50)	<0.001	1.66 (0.93–2.98)	0.088
**Marital status**				
Married (Ref.)	1		1	
Unmarried/separated/divorced/widowed	2.17 (1.67–2.84)	<0.001	1.68 (1.16–2.43)	**0.006**
**Employment status**				
Employed (Ref.)	1		1	
Unemployed	1.98 (1.44–2.72)	<0.001	1.72 (1.22–2.44)	**0.003**
**Ethnicity**				
Malays	0.56 (0.40–0.78)	0.001	0.67 (0.47–0.94)	**0.022**
Chinese	0.31 90.18–0.73)	<0.001	0.41 (0.20–0.83)	0.014
Indians	0.37 (0.18–0.73)	0.005	0.44 (0.19–1.00)	0.049
Other Bumiputras (Ref.)	1		1	
Other	0.64 (0.34–1.18)	0.153	0.74 (0.37–1.47)	0.385
**Education level**				
No formal education	6.05 (2.99–12.26)	<0.001	2.13 (1.02–4.45)	**0.045**
Primary education	3.46 (1.70–7.02)	0.001	1.62 (0.772–3.42)	0.199
Secondary education	1.85 (0.84–4.09)	0.127	1.22 (0.56–2.66)	0.619
Tertiary education (Ref.)	1		1	
**Income (MYR)**				
<1000	6.78 (3.23–14.20)	<0.001	3.67 (1.84–7.31)	**<0.001**
1000–1999	4.19 (1.81–9.71)	0.001	2.71 (1.23–5.94)	**0.014**
2000–2999	2.18 (0.70–6.83)	0.179	1.73 (0.60–5.02)	0.308
≥3000 (Ref.)	1		1	
**Smoking status**				
Non-smoker (Ref.)	1		1	
Ever smoker	1.60 (1.29–1.97)	<0.001	1.68 (1.23–2.29)	**0.001**

* Aged ≥60 years.

AOR: adjusted odds ratio; multiple logistic regression was performed to determine the association between smoking status and risk of depression, while adjusting for residential location, gender, age, ethnicity, marital status, education level, employment status, and monthly individual income in the logistic regression model. No interactions were found between the independent variables (p>0.05). Classification of table showed the model correctly predicts 88.8% of the cases. Receiver Operation Characteristic curve analysis showed area under the curve was 0.69. Bold indicates statistical significance at p<0.05. MYR: 100 Malaysian Ringgit about US$22.

## DISCUSSION

The present survey elucidates the smoking status and its relationship with depression among the elderly population of Malaysia. The present findings showed that ever smokers were more likely to be depressed than non-smokers among the elderly participants. Our finding concurs with a recent study completed in China affirming that among the middle-aged and older adults, short-term and moderate-term quitters showed an increased risk of enduring depression compared to non-smokers, irrespective of the time passed since cessation of smoking^13^. This survey explored depression in participants aged ≥45 years. Furthermore, based on pooled data among Canadians aged ≥65 years, cigarette smoking was constantly and progressively associated with depression^14^. Ever smokers comprised participants who were either former (ex) smokers and/or current smokers. It was postulated by Leventhal et al.^10^ that the reward associated with smoking was boosted by anhedonia which increased the pleasure-enhancing effects of smoking. Additionally, chronic smoking tended to perpetuate negative effects and anhedonia, which mutually curb attempts at cessation^15^.

Our analysis of marital status showed that single participants were more likely to be depressed. This result concurs with other studies across the globe^16-18^. According to Prince et al.^19^, single individuals in middle-age and elderly years often go through extended periods of feeling lonely, receiving less social support, and lacking confidence, which could potentially increase their vulnerability to depression. This study revealed that unemployed participants were more likely to be depressed than elderly participants. The results mirrored a study in Malaysia among elderly Malay participants^20^. Having a job boosts self-confidence and self-esteem, empowering individuals to take charge of their own future. These factors could potentially decrease their vulnerability to depression^21^. In terms of ethnicity, other bumiputras who were deemed minority groups were more likely to have depression compared to Malays and Chinese elderly participants. Comparable outcomes with their corresponding reasons were mentioned in two studies, which explored psychosocial issues among minority populations^22,23^. Our study showed that elderly participants without formal education were more likely to be depressed, with an estimated 2-fold increased risk of depression compared to those with tertiary education. This finding was consistent with another cross-sectional study conducted by Nyandar et al.^24^ involving 92 elderly people in Bali, Indonesia.

Income profiling demonstrated that elderly participants who had earned a monthly income of <2000 MYR were more likely to have depression compared with those who had earned ≥3000 MYR. This conclusion was also observed amongst the Brazilian population, using the same cut-off age definition for elderly persons as in our study but with a smaller participant strength of 1499 residents^25^.

### Strengths and limitations

The strength of the study lies in the premise that it represents the Malaysian population. However, we acknowledge several limitations in this study. Firstly, since the survey was cross-sectional in nature, it did not allow for cause-and-effect associations to be examined. Secondly, the inherent disadvantage of conducting a survey, which not only has recall bias but also becomes less reliable if responses are obtained under duress, especially in terms of time for completion by the participants concerned. Lastly, the use of a self-report tool, i.e. Geriatric Depression Scale, which is not a diagnostic tool, could create spurious results pertaining to under- or over-reporting of the association between smoking and depression.

## CONCLUSIONS

Ever smokers were more likely to have depression in the Malaysian elderly population. Public health awareness interventions, such as smoking cessation initiatives, are certainly a crucial tool and should be prioritized to promote mental well-being in this vulnerable population.

## Data Availability

The data supporting this research are available from the authors on reasonable request.
